# Compassionate Treatment of Brainstem Tumors with Boron Neutron Capture Therapy: A Case Series

**DOI:** 10.3390/life12040566

**Published:** 2022-04-10

**Authors:** Yi-Wei Chen, Yi-Yen Lee, Chun-Fu Lin, Ting-Yu Huang, Shih-Hung Ke, Pei-Fan Mu, Po-Shen Pan, Jen-Kun Chen, Tien-Li Lan, Ping-Chuan Hsu, Muh-Lii Liang, Hsin-Hung Chen, Feng-Chi Chang, Chih-Chun Wu, Shih-Chieh Lin, Jia-Cheng Lee, Shih-Kuan Chen, Hong-Ming Liu, Jinn-Jer Peir, Hui-Yu Tsai, Ko-Han Lin, Nan-Jing Peng, Kuan-Hsuan Chen, Yuan-Hung Wu, Yu-Mei Kang, Wan-Chin Yang, Shueh-Chun Liou, Wei-Hsuan Huang, Hiroki Tanaka, Tai-Tong Wong, Yee Chao, Fong-In Chou

**Affiliations:** 1 Faculty of Medicine, National Yang Ming Chiao Tung University, Taipei City 112304, Taiwan; yylee62@gmail.com (Y.-Y.L.); yhwu13@vghtpe.gov.tw (Y.-H.W.); mmmmeeeii@gmail.com (Y.-M.K.); wcyang3@vghtpe.gov.tw (W.-C.Y.); sjliou@vghtpe.gov.tw (S.-C.L.); 2 Department of Oncology, Taipei Veterans General Hospital, Taipei City 11217, Taiwan; yukatakids@hotmail.com (T.-L.L.); happyken176@gmail.com (J.-C.L.); skchen@vghtpe.gov.tw (S.-K.C.); ychao@vghtpe.gov.tw (Y.C.); 3 Department of Medical Imaging and Radiological Technology, Yuanpei University of Medical Technology, Hsinchu City 30015, Taiwan; 4 College of Nuclear Science, National Tsing-Hua University, Hsinchu City 300044, Taiwan; 5 Department of Neurosurgery, Taipei Veterans General Hospital, Taipei City 11217, Taiwan; chunfu0526@gmail.com (C.-F.L.); doc3379b@gmail.com (P.-C.H.); 6 Nursing Department, Taipei Veterans General Hospital, Taipei City 11217, Taiwan; llaa5405@yahoo.com.tw (T.-Y.H.); shke@vghtpe.gov.tw (S.-H.K.); 7 School of Nursing, National Yang Ming Chiao Tung University, Taipei City 112304, Taiwan; peifan@ym.edu.tw; 8 Department of Chemistry, Tamkang University, New Taipei City 251301, Taiwan; 138020@mail.tku.edu.tw; 9 Institute of Biomedical Engineering and Nanomedicine, National Health Research Institutes, Zhunan Town, Miaoli County 350, Taiwan; jkchen@nhri.edu.tw; 10 Department of Neurosurgery, Mackay Memorial Hospital, Taipei City 104217, Taiwan; liang4617@hotmail.com; 11 Department of Pediatric Neurosurgery, Taipei Veterans General Hospital, Taipei City 11217, Taiwan; hhchen3@vghtpe.gov.tw; 12 Department of Radiology, Taipei Veterans General Hospital, Taipei City 11217, Taiwan; fcchang374@gmail.com (F.-C.C.); ccwu6@vghtpe.gov.tw (C.-C.W.); 13 Department of Pathology and Laboratory Medicine, Taipei Veterans General Hospital, Taipei City 11217, Taiwan; diegolin@vghtpe.gov.tw; 14 Nuclear Science & Technology Development Department, National Tsing-Hua University, Hsinchu City 300044, Taiwan; hmliu@mx.nthu.edu.tw (H.-M.L.); jjpeir@mx.nthu.edu.tw (J.-J.P.); huiyutsai@mx.nthu.edu.tw (H.-Y.T.); fichou@mx.nthu.edu.tw (F.-I.C.); 15 Department of Nuclear Medicine, Taipei Veterans General Hospital, Taipei City 11217, Taiwan; khlin1979@gmail.com (K.-H.L.); njpeng@vghks.gov.tw (N.-J.P.); 16 Department of Pharmacy, School of Pharmaceutical Sciences, National Yang Ming Chiao Tung University, Hsinchu City 112304, Taiwan; khchen3@vghtpe.gov.tw; 17 Department of Pharmacy, Taipei Veterans General Hospital, Taipei City 11217, Taiwan; 18 Department of Radiation Oncology, Mackay Memorial Hospital, Taipei City 104217, Taiwan; hsuan782@gmail.com; 19 Institute for Integrated Radiation and Nuclear Science, Kyoto University, Kyoto 606-8501, Japan; tanaka.hiroki.3e@kyoto-u.ac.jp; 20 Department of Neurosurgery, Taipei Medical University, Taipei City 110301, Taiwan; ttwong99@gmail.com

**Keywords:** boron neutron capture therapy, brainstem tumor, diffuse midline glioma, compassionate use, bevacizumab

## Abstract

Brainstem tumors are heterogenous and cancerous glioma tumors arising from the midbrain, pons, and the medulla that are relatively common in children, accounting for 10% to 20% of all pediatric brain tumors. However, the prognosis of aggressive brainstem gliomas remains extremely poor despite aggressive treatment with chemotherapy and radiotherapy. That means there are many life-threatening patients who have exhausted all available treatment options and are beginning to face end-of-life stage. Therefore, the unique properties of highly selective heavy particle irradiation with boron neutron capture therapy (BNCT) may be well suited to prolong the lives of patients with end-stage brainstem gliomas. Herein, we report a case series of life-threatening patients with end-stage brainstem glioma who eligible for Emergency and Compassionate Use, in whom we performed a scheduled two fractions of salvage BNCT strategy with low treatment dosage each time. No patients experienced acute or late adverse events related to BNCT. There were 3 patients who relapsed after two fractionated BNCT treatment, characterized by younger age, lower T/N ratio, and receiving lower treatment dose. Therefore, two fractionated low-dose BNCT may be a promising treatment for end-stage brainstem tumors. For younger patients with low T/N ratios, more fractionated low-dose BNCT should be considered.

## 1. Introduction

Brainstem glioma is an intractable disease of the central nervous system, accounting for about 1% of adult brain tumors and up to 20% of pediatric brain tumors [1]. Since brainstem tumors are a heterogeneous group of low-grade and high-grade gliomas, the prognosis is markedly different [2]. Among all types of brainstem tumors, diffuse glioma is the most observed aggressive cancerous brain tumors in the world. According to the updated World Health Organization (WHO) 2021, diffuse gliomas are defined by both histology and molecular features into anaplastic oligodendroglioma, diffuse astrocytic gliomas, glioblastoma, and diffuse midline glioma [3]. Diffuse midline glioma, previously also known as diffuse intrinsic pontine gliomas, is a brain tumor that commonly grows in the pons of the brainstem, thalamus and spinal cord. Diffuse midline glioma predominantly occurs in children and have an extremely poor prognosis due to its infiltrative growth and inoperable feature, as well as high resistance to chemotherapy and radiotherapy [4,5]. H3 K27M-mutation can be used as an important diagnostic factor if biopsy is done for the midline glioma [6]. The overall survival (OS) is only 7.9–12.0 months despite the use of different radiation therapies (upfront conventionally fractionated, hyperfractionated, or hypofractionated radiotherapy) and chemotherapy (neoadjuvant chemotherapy prior to radiotherapy, adjuvant therapy, or chemoradiotherapy) [7,8,9]. Therefore, less than 10% of patients are alive 2 years after diagnosis [10,11]. For these patients in life-threatening or severely debilitating situation and have exhausted all available treatment options, it is necessary to use strategies other than chemoradiotherapy to prolong the survival of these patients as long as possible.

Unlike conventional radiation therapy, boron neutron capture therapy (BNCT) is a new modality of radiation therapy that uses epithermal neutron source to destroy boron-absorbing tumor cells with minimal damaging surrounding normal tissue [12]. Therefore, successful BNCT mainly depends on the selective accumulation of nonradioactive boron-10 atoms in tumor cells and thermal neutron beams. Currently, ^10^B-4-borono-L-phenylalanine (L-BPA) and sodium borocaptate (BSH) are common boron delivery agents in clinical use [13], and the sources of thermal neutrons are mainly from nuclear reactors or accelerator-based neutron sources [14,15]. Although BNCT is not been widely used in large-scale clinical trials, BNCT has recently achieved promising results in the treatment malignant brain tumors [16,17,18,19], recurrent head and neck cancer [20], and melanomas [21,22]. Even for recurrent glioblastoma and recurrent head and neck cancer, BNCT is comparable to other treatments, with a median survival of approximately 12 months [23]. Although BNCT has minimal damage to surrounding normal brain tissue and increasing radiation doses would theoretically improve efficacy, increasing BNCT dose may further increase the risk of radiation necrosis and symptomatic pseudo-progression. Furthermore, high-dose BNCT is even more unbearable for patients with end-stage recurrent malignant brain tumors, who are much weaker and more exhausted from past repeat treatments.

In this study, we report a group of critically ill patients with end-stage malignant brainstem tumors, in whom we performed a scheduled two fractions of salvage BNCT strategy with low treatment dosage each time. The aim of the present study was to report the clinical efficacy of scheduled two fractions of salvage BNCT for patients with histological proven brainstem glioma eligible for Emergency and Compassionate Use. Clinical outcomes as well as three representative cases of our scheduled two fractions of BNCT are summarized in the present study. In addition, the potential of a strategy of using multifractionated BNCT as a radiotherapeutic modality for compassionate treatment of brainstem tumors is discussed.

## 2. Materials and Methods

### 2.1. Patient Selection

From March 2017 to December 2021, critically ill patients with end-stage malignant brain tumors who comply the criteria for Emergency and Compassionate Use (Expanded Access Program) were identified in our hospital. After detailed explanation to the patient, BNCT was used as a salvage treatment for the severe and life-threatening brain tumors. Written informed consent was obtained from each patient or authorization from their parents. The BNCT treatment protocol was approved by the institutional review board of Taipei Veterans General Hospital, and each patient’s BNCT treatment was approved by the Taiwan Food and Drug Administration (TFDA). Demographics, clinical data, tumor characteristics, and BNCT parameters were extracted from the medical records, including age, sex, tumor diagnosis and distribution, Karnofsky performance status (KPS), tumor volume, blood boron concentration during neutron irradiation, tumor dose (mean, minimal and maximal), tumor response, toxicity and comorbidity as well as the presence of post-operative recurrence or radiation necrosis.

### 2.2. BNCT Procedure

In this study, the BNCT treatment protocol was modified from the protocol developed by Kyoto University, Japan [24,25,26]. Since the eligible patients were critically ill and in a life-threatening condition, the treatment strategy used in this study was a scheduled two fractions of low-dose BNCT with an interval of about 1 month. Before neutron irradiation, ^10^B-4-borono-L-phenylalanine (L-BPA) was administrated as described previously to estimate boron concentration by ^18^F-BPA-positron emission tomography (PET) [16,27]. The tumor-to-normal tissue (T/N) and tumor-to-blood (T/B) uptake ratios of each patient was calculated based on the maximum standardized uptake value (SUV) of the tumor lesion and adjacent normal tissue or left ventricle of heart. Patients received a 2-h infusion of L-BPA (200 mg/kg of body weight) prior to BNCT, followed by a continuous infusion of L-BPA (100 mg/kg) to maintain constant blood boron concentrations during neutron irradiation. The total dose of L-BPA infused per patient was 500 mg/kg. The source of epithermal neutron irradiation for BNCT was from Tsing-Hua Open-Pool Reactor (THOR) at National Tsing-Hua University. All patients received a scheduled two fractions of BNCT treatment. The irradiation dose for each patient was independently calculated based on the gross tumor volume (GTV) and blood boron concentration. The weighted Gy-E doses were calculated by multiplying the physical dose given by the relative biological effectiveness (RBE) or compound biological effectiveness (CBE). The CBE, an RBE-weighted dose of boron carrier, is used to assess the efficacy of the ^10^B(n, α)7 Li reaction. In this study, the CBE values of the ^10^B(n, α)7 Li reaction for the normal skin and brainstem tumor were 2.5 and 3.8, respectively. The RBE of thermal neutron and photons are 3.2 and 1.0, respectively. We have added above Gy-eq calculations in the Method section of the revised manuscript. After BNCT procedure, all patients were admitted to the ward of Neurosurgery Department of Taipei Veterans General Hospital and prescribed 5 mg of steroid to avoid neurological deterioration such as radiation necrosis. In addition, all patients received bevacizumab (Avastin) at doses of 5–10 mg/kg every two weeks after BNCT treatment to avoid the development of radiation necrosis [28,29]. All patients underwent MRI imaging 4 week after BNCT treatment to assess the first treatment response and every three-month interval for regular follow-up.

### 2.3. Response Evaluation

Magnetic resonance imaging (MRI) scan were performed before and after BNCT procedure to assess the radiologic response according to the response assessment in neuro-oncology criteria (RANO) [30]. A complete response (CR) was defined as the complete disappearance of the tumor within the brainstem after treatment. A partial response (PR) was defined as a ≥50% reduction in tumor volume after treatment (the largest cross-sectional tumor area) for at least 4 weeks, while a progressive disease (PD) was defined as a ≥25% increase in tumor volume after treatment. A stable disease (SD) was defined when the tumor volume after BNCT treatment was insufficient to be characterized as a CR, PR or PD. All patients were followed up every 1–3 months with MRI imaging and neurological examination. Toxicity after BNCT treatment were evaluated according to the National Cancer Institute (NCI) Common Terminology Criteria for Adverse Events (CTCAE) version 5.0 [31]. Time to relapse was defined as the time from the date of BNCT treatment to the time of relapse.

## 3. Results

A total of 7 critical ill patients who met Compassionate Use criteria were treated with salvage BNCT, including 4 males and 3 females (Table 1). Of the 7 patients, 4 were children under the age of 12. One patient was diagnosed with ganglioglioma, one low grade glioma compatible with pleomorfic xantoastrocitoma (WHO grade II), and one glioblastoma. Four patients were diagnosed with high-grade malignant gliomas, one of which was astrocytoma progressing to high-grade glioma, and two of which were diffuse midline glioma. In order to reduce the adverse effects, all patients underwent two fractions of low-dose BNCT (5–10 Gy-E for each fraction) procedures by the same experienced BNCT team with an interval of about 1 month. The median tumor dose of the first fraction and second fraction were 7.22 Gy-E and 6.62 Gy-E, respectively. The median blood boron concentrations during the first and second neutron irradiation were 24.83 ppm and 26.95 ppm, respectively. The mean T/N and T/B ratio in the first BNCT fraction were 3.02 and 3.41, respectively, while in the second fraction were 2.77 and 2.76, respectively. The length of BNCT procedure was all successfully completed within 30 min. After two fractions of BNCT treatment, only some patients experienced mild acute adverse events, such as radiation dermatitis and alopecia, and these patients were treated conservatively with analgesics and topical steroids. No patients developed grade 4 acute or late adverse events. Only one patient with high-grade diffuse midline glioma showed radiation necrosis, which was assessed as likely due to hypofractionated radiation therapy prior to BNCT. The MRI scans showed CR in 1 case, PR in 5 cases, and SD in 1 case. No patient’s status was characterized as PD after BNCT. Of the three patients who had lower T/N ratio (below median values) and received lower treatment doses (below median values) at the first and second fractions, they all relapsed at 1, 12, and 13 months after BNCT treatment.

### 3.1. Representative Cases

#### 3.1.1. Case 1 (Patient #5)

The patient is a 6-year-old boy who first presented with limited movement of the eyes accompanied by marked drowsiness in March 2020. The patient was diagnosed via MRI with a diffuse midline glioma in the right midbrain. He began a hypofractionated split-course of radiation therapy (39 Gy, 13 fractions) at the end of March 2020 in foreign country and received chemotherapy in July 2020.

**Figure 1:**
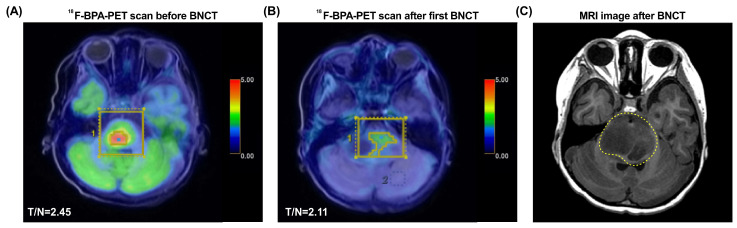
^18^F-BPA-PET and MRI images of Case 1 (patient #5). (**A**) The ^18^F-BPA-PET reveals the location of tumor activity before BNCT treatment. (**B**) The ^18^F-BPA-PET reveals the location of tumor with reduced tumor activity after first procedure of BNCT treatment. (**C**) The follow-up MRI image of the patient shows radiation necrosis in the fourth ventricle (dashed yellow line). The yellow squares indicate the tumor location before and after BNCT treatment.

Because of the inadequacy of any available standard treatment options after diagnosis, the patient was enrolled in our TFDA-approved Expanded Access Program for compassionate use treatment with BNCT. After examination by the BNCT team at Taipei Veterans General Hospital, Taiwan, the brain images showed a tumor volume of 8.77 mL, and the L-BPA uptake study showed a T/N ratio of 2.45 (Figure 1A). The patient received a scheduled two fractions of salvage BNCT procedure on 17 July 2020 and 14 August 2020. The mean tumor dose in the first BNCT treatment was 7.22 Gy-E, with a minimal tumor dose of 4.19 Gy-E and a maximal tumor dose of 11.54 Gy-E. The boron concentration in blood at the time of first BNCT were 24.19 ppm. The ^18^F-BPA-PET examination about one month after the first BNCT showed that the T/N ratio decreased to 2.11 (Figure 1B). The mean tumor dose of second BNCT was 5.76 Gy-E (minimal: 3.38 Gy-E and maximal: 9.40 Gy-E), and the blood boron concentration was 24.62 ppm. The follow-up examination revealed a PR. However, after two fractions of low doses of BNCT procedures, the tumor still progressively enlarged at the pons (4.2 cm × 3.1 cm × 2.4 cm), with marked perifocal edema. The time to relapse was 61 days. Follow-up MRI of the brain revealed radiation necrosis in the fourth ventricle (Figure 1C), which caused acute obstructive hydrocephalus. The patient’s symptoms still progressed, including limited extraocular motility (EOM) with bilateral esotropia, decreased facial expression, blurred speech, dysphagia, left-sided hemiparesis, mild dysuria, and constipation. A right ventriculoperitoneal shunt was placed and mannitol and dexamethasone were administrated to resolve the elevated intracranial pressure. The patient survived 4.9 months after BNCT and died of disease progression.

#### 3.1.2. Case 2 (Patient #3)

The 49-year-old Caucasian women was diagnosed with pilocytic astrocytoma (WHO grade I) in 2016, a slow-growing primary central nervous system tumor. She underwent a total of two craniotomies and conventional radiotherapy, and two Gamma Knife radiosurgery in Spain. In addition, the patient received four cycles of temozolomide treatment (5 consecutive days of a 28-day cycle). However, the pathology, diagnosed in 2018, has been upgraded with anaplastic component. The patient experienced treatment failure after further receiving 2 cycles of procarbazine, lomustine, and vincristine (PCV) regimen and 1 cycle of temozolomide regimen before Gamma Knife radiotherapy. As multiple treatment strategies were ineffective, the patient came to our ward in 2019 to seek the possibility of salvage BNCT treatment.

**Figure 2:**
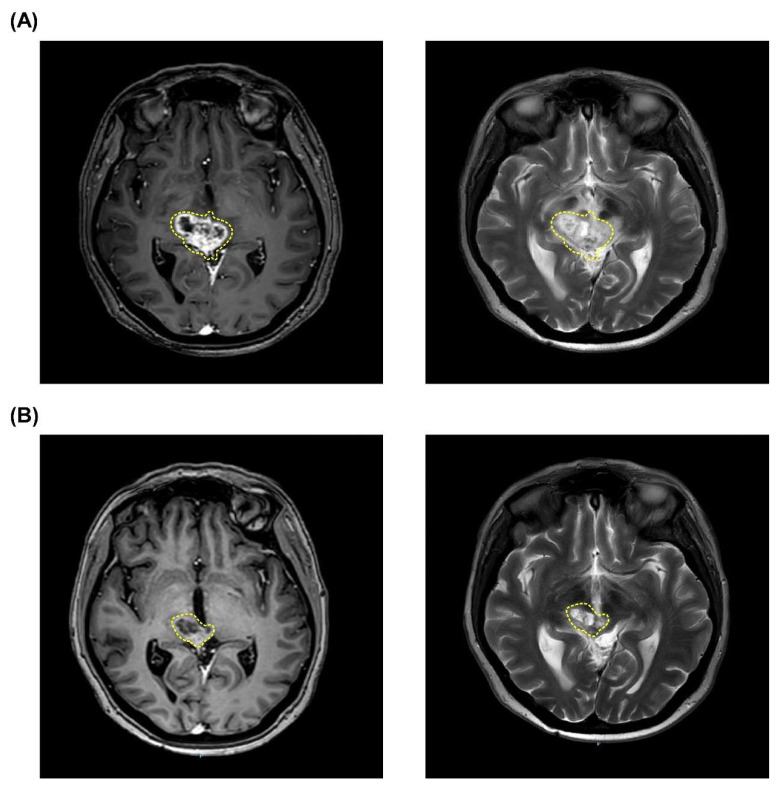
MRI images of tumor shrinkage of Case 2 (patient #3). Tumor volume before (**A**) and after (**B**) a scheduled two fractions of salvage BNCT procedures. The pleomorphic xanthoastrocytoma in the images is indicated by yellow dashed circles.

After further examination, the tumor had been developed into a pleomorphic xanthoastrocytoma (WHO grade II) with recurrence over the brainstem (Figure 2A). After a detailed evaluation and approval of TFDA emergency and compassionate use, two fractions of BNCT procedures were scheduled and conducted. The ^18^F-BPA-PET showed that the T/N and T/B ratios in the first BNCT were 2.46 and 2.87, respectively, and the T/N and T/B ratios in the second BNCT were 2.34 and 2.39, respectively. The mean, minimal, and maximal brain tumor dose in the first BNCT procedure were 6.79, 5.42, and 9.16 Gy-E, respectively. The mean, maximal, and minimal brain tumor dose in the second BNCT procedure were 6.62, 5.81, and 8.01 Gy-E, respectively. Blood boron concentrations during the first and second BNCT procedures were 41.13 ppm and 27.41 ppm, respectively. No severe acute or late toxicity observed. Follow-up examinations showed that the patient achieved a PR (Figure 2B) and has not relapsed until now. The patient with life-threatening pleomorphic xanthoastrocytoma survives more than 662 days after two fractions of low doses BNCT procedures.

#### 3.1.3. Case 3 (Patient #7)

The male patient first presented with headaches and dizziness since 2019 and was initially diagnosed with a brainstem anaplastic astrocytoma (WHO grade 3) at the age of 30. He underwent tumor resection through the far-lateral transcondylar approach and thereafter treated by radiotherapy (54 Gy, 30 fractions) and chemotherapy in August 2019. However, follow-up MRI showed that the tumor had recurred two years later, and the tumor was transformed as are recurrent glioblastoma after biopsy evaluation. Because this critically ill patient was in a life-threatening condition and has no other treatment options, the patient is eligible for compassionate use of BNCT.

**Figure 3:**
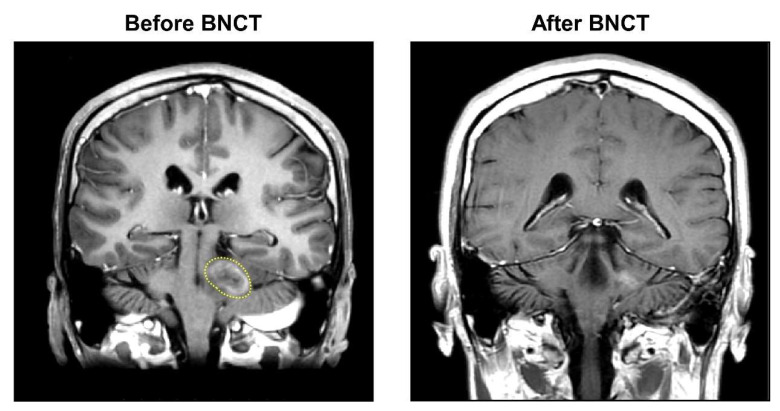
MRI images of tumor shrinkage of Case 3 (patient #7). After a scheduled two fractions of salvage BNCT procedures, the patient achieved a CR with obvious tumor shrinkage. The yellow dashed line marks the malignant glioblastoma.

The ^18^F-BPA-PET examination showed therapeutically adequate boron accumulation in the tumor tissues, with a T/N ratio of 3.83. The patient was treated by BNCT one in November 2021 and once again in December 2021. The mean tumor dose in the first and second BNCT were 10.95 Gy-E and 9.68 Gy-E, respectively. The blood boron concentration during the first and second neutron irradiation were maintained at 22.85 ppm and 26.95 ppm, respectively. The patient achieved a CR after two fractionated BNCT therapies (Figure 3). No acute and late adverse events were observed. The patient was doing well at the time of manuscript submission, with no tumor progression and recurrence.

## 4. Discussion

In our case series, BNCT showed promise for patients with end-stage brainstem tumors who have exhausted other effective treatment options. Regardless of tumor volume, survival in these life-threatening patients can be prolonged as long as possible with our scheduled two fractionated and low-dose BNCT regimen. With the exception of one patient whose necrosis was attributable to prior treatment, none of the other patients experienced severe acute and late adverse events after our BNCT regimen. Furthermore, we found that patients who relapsed after BNCT had three characteristics, including younger age (<12 years), had a lower T/N ratio (below median: <2.46), and treated with lower treatment doses (all below median: first < 7.22 Gy-E and second < 6.62 Gy-E). Remarkably, we observed 2 long-term survivor who did not develop a relapse more than 18 months, despite being diagnosed with end-stage brainstem tumors and in a life-threatening condition prior to BNCT treatment. Therefore, a low-dose two-fractioned BNCT procedure is effective in patients with end-stage brainstem tumors who are eligible for compassionate use without severe acute or late toxicity.

Brainstem gliomas remains a challenging tumor type due to their highly heterogenous clinical features, with many clinical trials failing to improve survival. Several studies have found that older age, lower KPS, higher tumor grade and appearance of necrosis are unfavorable prognostic factors for survival [32,33,34]. In our case series, however, age, KPS, and tumor grade did not appear to affect the efficacy of BNCT. Conversely, younger patients with end-stage brainstem tumors may be prone to relapse. All three young patients with diffuse entities in our study relapsed after two fractioned BNCT treatments (Patients #2, #5, and #6). We found that the common feature of the three relapsed patients was a low T/N ratio during the ^18^F-BPA-PET examination, suggesting that the tumors of these young patients absorbed little boron. In theory, inadequate boron in the tumor cells would not be able to capture sufficient epithermal neutron beam, resulting in low α-particle generation that would not be able to destroy the tumor cells [12]. A similar feature of low boron uptake in pediatric brain tumors was found in our previous study [35], in which 70% of pediatric brain tumor patients had a T/N ratio ≤ 2.8 during preoperative FBPA-PET examination. On the other hand, allow dose-fractionation strategy was implemented in these end-stage brainstem tumors to avoid severe adverse events and maintain quality of life. Since the features of low T/N ratio in children and the low dose of BNCT given may be the main reasons for tumor relapse, we recommend to increase the fractionations of BNCT under the low-dose strategy to improve the therapeutic effect of BNCT on pediatric brainstem tumors.

Patients with brainstem gliomas may begin to face the end-of-life stage when tumor progression persists and further tumor treatment is not an option. Therefore, the purpose of our salvage BNCT is not only to prolong the life as long as possible, but also to maintain quality of life with no or low adverse effects. This is also why we employ a fractionated and low-dose BNCT approach to reduce adverse events when considering the declining health status of patients with end-stage brainstem tumors. Furthermore, multiple fractionated BNCT can not only deliver curative doses to the tumors, but also keep the delivered dose below tolerance level. Haginomori et al. in 2009 suggested that planned fractionated BNCT is also effective for patients with inoperative recurrent squamous cell carcinoma in the temporal bone [36]. The advantage of 2 planned fractionated BNCTs is that the first BNCT procedure targets the relatively superficial part of the tumor, while the second BNCT procedure one month later plans to target the deeper residual part of the tumor. Another study in Taiwan also showed that 2 planned fractionated BNCTs with adaptive dose prescription was also effective and safe in the treatment of locally recurrent head and neck cancer [37]. In our case series, all patients received 2 planned fractionated BNCTs, and no patients had severe acute and late adverse events.

The only patient (Case 1) who developed radiation necrosis was unrelated to current BNCT therapy, as this patient had received multiple high-dose radiation therapies (39 Gy in 13 fractions) in the foreign country three months prior to BNCT. Based on the Oxford Centre for Evidence-based Medicine [38] and the Radiotherapy Dose Fractionation guideline for pediatric cancer [39], conventional fractionated radiotherapy with a dose of 54 Gy in 30 fractions over six weeks is recommended for the treatment of pediatric brainstem glioma. Although increasing the radiation dose from 54 to 59.4 Gy for children after surgery is thought to obtain more durable local control [40], the hypofractionated radiotherapy boost approach may not be appropriate for children with end-stage brainstem tumors, possibly because of the development of radioresistance. In our clinical practice of BNCT, we suggested that more fractionated low-dose BNCT should be conducted for end-stage brainstem tumor patients.

Based on our current treatment series, the low-dose fractionation strategy of salvage BNCT is a powerful weapon against brainstem tumors, with the additional advantage of avoiding serious adverse events. However, children need to remain still without anesthesia for about 20 min to allow thermal neutrons to irradiate the tumor evenly, which is a big challenge. Fortunately, our previous study showed that recreational strategies such as watching cartoons projected on the ceiling of the treatment room can attract children’s attention and reduce anxiety in pediatric brain tumor patients during external beam radiotherapy [38]. In addition, praising or giving a favorite toy after treatment as a reinforcement strategy can enhance their willingness to participate in next treatment. On the other hand, for pediatric brain tumor patients who needs treatment, their parents often face tremendous stress and pain, conflict and uncertainty while making illness-related decisions [41]. Recently, the shared decision-making process in the medical system has received increasing attention, that is, clinicians and patients communicate with each other to share medical information and family status when facing with various medical decision [42]. Since the shared decision-making process is a continuous and dynamic process, integrating family communication with the medical team can not only construct the decision-making process step-by-step, but also help nurse provide individual individualized care for each pediatric brain tumor patient [43]. Therefore, two nonpharmacologic approaches are conducted in our BNCT procedures, including pre-treatment interviews with the doctors to reduce parental anxiety, to improve the understanding of the disease and BNCT procedures, and to improve doctor-patient interaction, and the use of audiovisual animations in the perioperative period to reduce children’s anxiety and distract their consciousness.

There are some limitations in this study. First, the sample size in this study is small and insufficient to provide further statistical analysis, such as overall survival, progression-free survival, and relapse-free survival. This is because all patients were end-stage brainstem cancer patients and in a life-threatening state, and each BNCT treatment need to be applied one by one according to the principle of Compassionate Use. Second, this study did not record the improvement of the quality of life of these patients (or even their parents) after BNCT. In future prospective studies, exploring the improvement of quality of life and home nursing care in end-stage brainstem tumor patients after BNCT are also important indicators that need to be further considered.

## 5. Conclusions

Salvage BNCT based on a low-dose fractionation strategy appears to be a safe and efficacious modality of treatment for patients with end-stage brainstem gliomas who eligible for Compassionate Use. For pediatric vulnerable brainstem tumor patients, due to their low T/N ratio nature, more fractionated low-dose BNCT procedures should be considered in the future when the T/N ratio cannot be improved.

## Figures and Tables

**Table 1:** Demographic characteristics, BNCT parameter, and treatment outcomes.

Patient	Age	Sex	Diagnosis	Distribution	KPS	Tumor Volume (mL)	1st BNCT						2nd BNCT						Response	Toxicity	Relapse
							T/N	T/B	Boron (ppm)	Tumor Dose (Gy-E)			T/N	T/B	Boron (ppm)	Tumor Dose (Gy-E)					
										Mean	Min	Max				Mean	Min	Max			
1	34	F	High-grade glioma (astrocytoma)	Diffuse	80	13.60	3.00	2.54	28.02	12.33	10.12	17.74	2.09	1.78	27.16	7.75	4.29	14.45	PR	No	No
2	8	M	High-grade glioma	Diffuse	50	35.60	1.94	1.38	19.80	6.30	5.02	7.48	1.52	1.52	20.22	6.58	5.46	8.26	SD	No	Yes
3	49	F	Pleomorfic xantoastrocitoma	Focal	60	17.72	2.46	2.87	41.13	6.79	5.42	9.17	2.34	2.39	27.41	6.62	5.81	8.01	PR	No	No
4	12	M	Ganglioglioma	Focal	80	25.73	5.43	7.02	24.83	9.35	5.30	14.96	5.43	7.02	29.02	9.89	4.67	17.17	PR	No	No
5	6	M	High-grade diffuse midline glioma	Diffuse	70	8.77	2.45	5.53	24.19	7.22	4.19	11.54	2.11	2.11	24.62	5.76	3.38	9.40	PR	Radiation necrosis ^†^	Yes
6	9	F	High-grade diffuse midline glioma	Diffuse	60	15.11	2.04	2.30	28.26	5.02	2.25	9.05	2.04	2.30	25.57	5.00	2.81	8.06	PR	No	Yes
7	30	M	Glioblastoma	Focal	70	1.59	3.83	2.22	22.85	10.95	7.24	18.20	3.83	2.22	26.95	9.68	6.38	16.17	CR	No	No

^†^ Radiation necrosis may be caused by previous hypofractionation radiotherapy regimen. Abbreviation: T/N, tumor-to-normal tissue uptake ratio; T/B, tumor-to-blood uptake ratio; KPS, Karnofsky performance status; CR, complete response; PR, partial response; SD, stable disease.

## Data Availability

The data presented in this study are available on request from the corresponding author.
